# Annotated ESTs from various tissues of the brown planthopper *Nilaparvata lugens*: A genomic resource for studying agricultural pests

**DOI:** 10.1186/1471-2164-9-117

**Published:** 2008-03-03

**Authors:** Hiroaki Noda, Sawako Kawai, Yoko Koizumi, Kageaki Matsui, Qiang Zhang, Shigetoyo Furukawa, Michihiko Shimomura, Kazuei Mita

**Affiliations:** 1 National Institute of Agrobiological Sciences, Owashi, Tsukuba, Ibaraki 305-8634, Japan; 2 Mitsubishi Space Software Co. Ltd, Takezono, Tsukuba, Ibaraki 305-0032, Japan; 3 Magnabeat Inc., Goi-kaigan, Ichihara, Chiba 209-8551, Japan

## Abstract

**Background:**

The brown planthopper (BPH), *Nilaparvata lugens *(Hemiptera, Delphacidae), is a serious insect pests of rice plants. Major means of BPH control are application of agricultural chemicals and cultivation of BPH resistant rice varieties. Nevertheless, BPH strains that are resistant to agricultural chemicals have developed, and BPH strains have appeared that are virulent against the resistant rice varieties. Expressed sequence tag (EST) analysis and related applications are useful to elucidate the mechanisms of resistance and virulence and to reveal physiological aspects of this non-model insect, with its poorly understood genetic background.

**Results:**

More than 37,000 high-quality ESTs, excluding sequences of mitochondrial genome, microbial genomes, and rDNA, have been produced from 18 libraries of various BPH tissues and stages. About 10,200 clusters have been made from whole EST sequences, with average EST size of 627 bp. Among the top ten most abundantly expressed genes, three are unique and show no homology in BLAST searches. The actin gene was highly expressed in BPH, especially in the thorax. Tissue-specifically expressed genes were extracted based on the expression frequency among the libraries. An EST database is available at our web site.

**Conclusion:**

The EST library will provide useful information for transcriptional analyses, proteomic analyses, and gene functional analyses of BPH. Moreover, specific genes for hemimetabolous insects will be identified. The microarray fabricated based on the EST information will be useful for finding genes related to agricultural and biological problems related to this pest.

## Background

The brown planthopper (BPH, *Nilaparvata lugens*) attacks rice plant and sucks fluid from the vascular bundle. Heavy infestation in the field causes *hopper burn*: complete death of the rice plants. In addition to sucking damage, BPH is a vector for the rice ragged stunt virus (RRSV) and rice grassy stunt virus (RGSV) [1]. Attack of this pest has caused intermittent serious famines in the East Asia since ancient times; it became conspicuous in the Southeast Asia after the so-called Green Revolution of the 1960s. Actually, BPH shows two wing forms, long (macropterous) and short (brachypterous) ones, in its adult stage. Macropterous adults fly long distances and invade rice-growing area. Most females of the subsequent generation of the immigrants are brachypterous – adapted for reproduction – and produce numerous offspring in rice fields. The wing dimorphism is induced by environmental stimuli during nymphal stages. These biological properties of BPH are closely related to BPH distribution, BPH reproduction, and rice plant damage.

Control methods of BPH primarily include the application of insecticides; various chemicals, organo-phosphorous, carbamate, pyrethroid, and neonicotinoid insecticides have been used in the field. However, insecticide application poses two problems: appearance of insecticide-resistant planthoppers [2-4]; and stimulation of planthopper propagation by insecticide treatment, known as resurgence [5,6]. Planthopper-resistant varieties of rice, which are widely cultivated in Asian countries, are also used as means of controlling BPH. However, cultivation of the planthopper-resistant varieties has engendered new virulent strains of BPH that can attack the resistant varieties [7]. Elucidation of rice plants' genes that increase resistance against planthoppers and BPH virulence mechanisms against the resistant rice plants have become important tasks. The former subject, that of resistance genes of rice plants, is studied extensively based on positional cloning, so-called map-based cloning procedures [8-10]. The latter problem, appearance of virulent strains of planthopper, awaits precise studies based on recent molecular approaches.

Rice planthoppers have been studied from ecological and physiological viewpoints but they have remained poorly investigated in genetic terms. A valid method for introducing molecular studies into insects without a genetic background is construction of an EST database. Information of genes working in various tissues at various growth stages is extremely useful for molecular functional study in biology. The ESTs of many insects have been accumulated; those of target insects have expanded to include agricultural pests, such as the migratory locust [11], aphids [12-14], whitefly [15], and armyworm [16]. Generation of ESTs in these agricultural pests is a first step to development of genomic resources for helping to solve agricultural problems.

The order Hemiptera, which includes *Nilaparvata lugens*, comprises two suborders: Heteroptera and Homoptera. Genome studies of Heteroptera are poor and a small-scale EST study have been reported [17], although large scale ESTs have been generated from homopteran insects, the pea aphid *Acyrthosiphon pisum *[14], the whitefly *Bemisia tabaci *[15], the green peach aphid *Myzus persicae *[18], the cotton aphid *Aphis gossypii *(>8,300 ESTs), and the glassy-winged sharpshooter *Homalodisca coagulata *(>20,000 ESTs). In addition, EST studies have been reported for the cereal aphid *Rhopalosiphum padi *[13] and the brown citrus aphid *Toxoptera citricida *[12]. A whole genome sequencing project is undertaken in a model insect *A. pisum*.

The present study was designed to acquire elusive EST data from different tissues and to provide data for discovery and identification of genes involved in fundamental biological phenomena and agricultural problems related to BPH. We also intend to undertake microarray-based studies in this planthopper species. We have collected about 37,000 high-quality EST data and more than 10,000 clustered unigenes from various tissues of this important pest. The datasets are available at DNA databases under the following accession numbers: [DDBJ/EMBL/GenBank: DB820956–DB858077] and at the website for BPH cDNA [19].

## Results and Discussion

### Quality control of cDNA libraries

**Figure 1:**
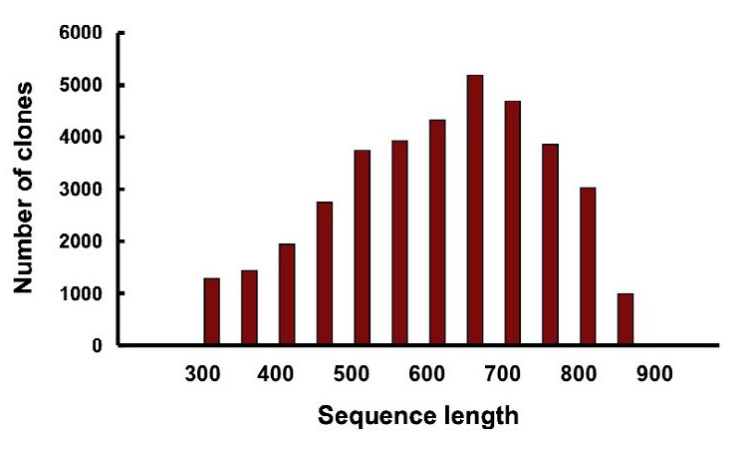
EST size distribution. ESTs of more than 299 bp were grouped by each 50 bp.

We produced 18 libraries (Table 1) from 15 different tissues or stages of the brown planthopper (BPH) to obtain various transcripts and to extract tissue-specifically expressed genes or planthopper-specific genes from the libraries. Two vectors, lambda-ZAP II and pGEM, were used for construction of the cDNA libraries. The former phage libraries were used in the early stages of study; later we constructed pGEM libraries using SMART technology. Single-path sequencing of the cDNA, which provided the sequence from the 5' region of mRNA, was done in each library. In all, more than 50,000 clones were processed, and 42,901 high-quality sequences of more than 299 bp long were selected. Vector sequences franking with the EST sequences were trimmed manually. Sequence contamination of lambda-ZAP II vector or lambda phage (87 clones) and the host organism *Escherichia coli *(130 clones) were deleted from the libraries based on in-house BLAST searches. The sequences of three major commensalistic viruses in the planthopper, NLRV [20,21], NLCXV [22], and Hi-PV [22,23], of which numbers in the ESTs were 0, 2, and 574 clones, respectively, were discarded. The Hi-PV sequences were mainly found from libraries of the female (MA) and male (MB) midgut, which is a major infection and propagation site of Hi-PV [24]. Mitochondrial sequences, which formed about 11% of the selected sequences (4,653/42,108), were subsequently eliminated. Ribosomal RNA (214 clones) and retrotransposon R2 (90 clones) sequences were also eliminated from the remaining ESTs. The resultant 37,144 sequences were further checked for short vector sequences using NCBI VecScreen. Chimeric sequences, which include two or more different mRNA sequences in an EST sequence, were then surveyed. ESTs were clustered based on the result of BLASTN (95% identity, 80% cover ratio); positional distributions of ESTs in each group were shown on a computer screen using the graphical overview of NCBI BLAST. The chimeric sequences were checked manually to determine whether two contigs were included in an assembled group or not. The chimeric sequences were usually found as those connecting two EST contigs or those having sequence extension to other members. Some, but not all, chimeric sequences appear to have been deleted. Finally, 37,122 cleaned sequences were deposited in the BPH database. Some ESTs, however, might retain several extra nucleotides at the 5' end. The average length of all ESTs was 626.7 bp; the range of most abundant sizes of sequences was 651–700 bp (Fig. 1). Ambiguous nucleotides, which are shown in "N", were 0.065% on average (0.4 bp per each EST) among all EST sequences (Table 1).

**Table 1:** List of the EST database of *Nilaparvata lugens*

Library	Tissue/sex/stage	Vector used	No. of clones	Average length (bp)	%"N"	No. of clusters
AA	Abdomen, female, 0–1 day	ZAP	609	589.7	0.014	435
AB	Abdomen, female, 0–1 day	ZAP	1027	601.9	0.011	667
AC	Abdomen, male, 0–1 day	ZAP	588	599.7	0.022	493
AD	Abdomen, male, 0–1 day	ZAP	967	594.9	0.102	726
EA	Egg, 0–1.5 day	pGEM	3726	652.0	0.051	2371
EB	Egg, 3–5 day	pGEM	2055	576.3	0.076	941
HA	Head, female, 0–1 day	ZAP	777	520.6	0.018	111
HT	Head-thorax, male, 0–1 day	ZAP	2555	673.9	0.070	1132
MA	Midgut, female, 0–5 day	pGEM	3594	637.5	0.051	1252
MB	Midgut, male, 0–5 day	pGEM	2095	606.4	0.117	847
NA	Whole nymph, 1st instar	pGEM	3269	633.8	0.096	1714
NB	Whole nymph, 4th instar	ZAP	3710	624.7	0.058	1749
OA	Ovary, 0–1 day	ZAP	967	511.5	0.119	689
OB	Ovary, 4–5 day	ZAP	822	601.9	0.028	329
OC	Ovary, 4–5 day	ZAP	2225	604.4	0.079	989
SG	Salivary glands, female, 0–5 day	pGEM	2383	640.8	0.083	723
TA	Testis, 0–1 day	ZAP	1525	575.9	0.045	895
TH	Thorax, 0–1 day	ZAP	4228	693.1	0.029	1657
						
Total			37122	626.7	0.065	10261

ZAP, lambda ZAP II (pBluescript) phage vector; pGEM, pGEM-t plasmid vector; %"N", percentage of ambiguous nucleotides. Ovary and testis includes the accessory glands of the reproductive organs. Clustering was performed using CLOBB [25]. ESTs are deposited in the DNA databases under accession numbers DB20956–DB858077 (37,122 entries).

### EST clustering

**Figure 2:**
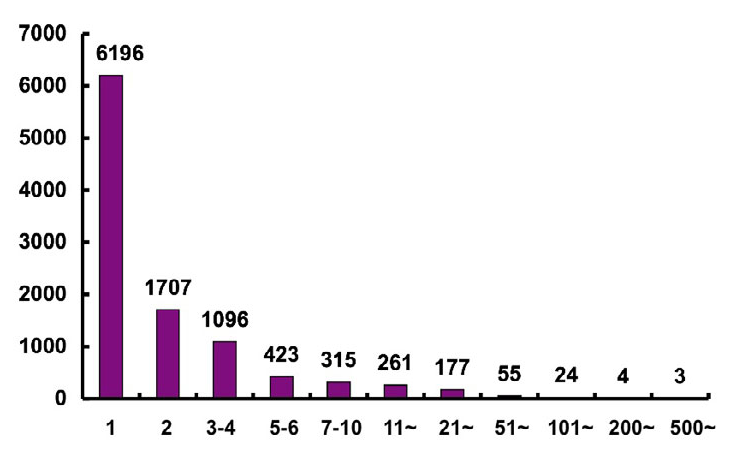
Relation between the number of clusters (vertical) and cluster size (horizontal). Clusters were calculated using the CLOBB clustering algorithm [25]. The number of clusters (unigenes) was 10,261, with 6,196 singletons.

All cleaned EST sequences were subjected to clustering using two clustering protocols, Cluster on the basis of BLAST similarity (CLOBB) [25] and Method of Combined BLAST and PhredPhrap (CBP) [26]. Mainly, CLOBB was used but the results of clustering generally differ among protocols. For that reason, two clustering methods were compared. First, CLOBB analyses were performed using parameters of 95% identity and 50 bp coverage (Table 1), resulting in 10,261 clusters (6,196 singletons and 4,065 contigs) (Fig. 2). Sequence assembly of the members of each contig, however, sometimes showed sequence variation (data not shown). This seems to be attributable to the following reasons: planthopper specimens collected from the stock culture possess heterogeneity in genome sequences among individuals. In addition, more than one similar gene was encoded on the genome. The second protocol, CBP, created 12,303 clusters (8,053 singletons and 4,250 contigs) using the parameters in additive manner, >97% identity and >90 bp coverage [26]. Actually, CBP tended to separate the members of each contig, whereas CLOBB sometimes included much sequence variation in each contig. A BPH database [19] was constructed based on CBP clustering results.

### Comparison of two cloning systems for EST analyses

In the EST project for small insects, the whole body is often used as the starting material because it is difficult to obtain a sufficient amount of total RNA or mRNA otherwise. In fact, female adult BPHs are about 4-mm long; the RNA amount from the dissected small tissue is therefore insufficient for a phage library. We used two cloning systems: lambda ZAP II phage (phage library) and pGEM plasmid (plasmid library). In general, the phage libraries are better than the plasmid libraries because the former can clone a larger insert DNA and create a larger number of clones than the latter. However, cloning into the pGEM plasmid is easier than that into ZAP II phage; moreover, it can be performed using a smaller amount of mRNA sample. Here, we compared the quality of the plasmid libraries with the phage libraries in two respects: the rate of vector contamination and the unigene ratio in each library.

First, contamination of cloning vectors in the EST sequences was compared in 42,901 ESTs (23,684 ESTs in phage libraries and 19,217 in plasmid libraries) using in-house BLAST, as described above. In the phage libraries, 87 lambda phage sequences were found, although no pGEM vector sequences were detected in the plasmid libraries. Host bacterial genome contamination was found in 129 clones in the phage libraries and one in the plasmid libraries. The plasmid library is excellent from the viewpoint of contamination of vector sequences and host bacterial genome sequences.

**Figure 3:**
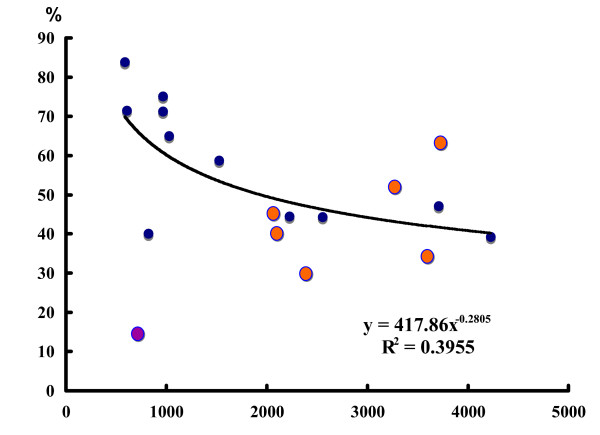
Comparison of two different vector libraries in the ratio of unigenes. The unigene rate against the number of ESTs analyzed was plotted for 18 different tissue libraries. Vertical axis, percentage (number of unigenes/the number of ESTs analyzed); horizontal axis, the number of ESTs analyzed in each library. Blue, the libraries of lambda-ZAP II; orange, the libraries of pGEM. The CLOBB algorithm was used for unigene calculation. The HA (female head) library, shown in violet, was excluded from calculation of the regression curve because this library showed an extremely low unigene rate against the number of ESTs (111/777 = 0.143).

Second, unigene ratios were calculated against the number of ESTs in each library. In the plasmid libraries, cDNA was prepared using SMART technology (Clontech Laboratories, Inc. USA), which uses PCR amplification. Therefore, the possibility is considered that biased amplification reduces the variety of cDNA. This might consequently decrease the ratio of unigenes in ESTs. Figure 3 shows the relation between the unigene ratio and the number of analyzed ESTs. The vertical axis shows the ratio (number of unigenes/number of ESTs), and the horizontal axis shows the number of ESTs analyzed. The number of unigenes corresponding to the number of clusters was calculated using CLOBB (Table 1). No marked low unigene ratio in the plasmid library was observed; rather, the ratio was varied among libraries (Fig. 3). One phage library (HA, female head) showed an extremely low unigene ratio (0.143), which was excluded for calculation of regression curve in Fig. 3. No remarkable disadvantage was observed in the plasmid libraries in terms of the unigene ratio. However, consideration of biased amplification and of polymerase amplification error is required for EST sequences of the plasmid libraries. A low amplification cycle number is preferable for construction of plasmid libraries.

### Abundantly expressed genes

Most abundantly expressed genes were extracted from the 37,122 ESTs based on the CLOBB clustering results. The contigs that included the top ten members are presented in Table 2. Contigs containing many ESTs were simply selected from the top, although the numbers of ESTs differ among libraries, which appears to affect the ranking of highly expressed genes.

**Table 2:** Top ten genes frequently appearing in the planthopper whole EST library

No.	Representative	No. of members	BLASTN	BLASTX
1	AA0004	727	actin	actin
2	EA0106	577	n.h.	n.h.
3	HA0070	542	mucin-like protein	mucin-like protein
4	AA0350	282	trypsin-like protease	trypsin-like protease
5	AA0116	254	ribosomal protein S2	ribosomal protein S2
6	AA0013	238	vitellogenin	vitellogenin
7	AA0383	228	n.h.	low homology
8	AA0260	186	myosin light chain	myosin light chain
9	AA0046	171	enolase	enolase
9	AB0581	171	n.h.	n.h.

The number of members was calculated using the CLOBB clustering algorithm. Genes are shown by the EST number that is representative of the cluster members. AA0383 is the same cluster member of OC2756, which is highly expressed in the ovary, as shown in Table 4. "n.h." denotes no homology in BLAST searches (see Gene annotation in *Methods*).

The actin gene was the most abundantly expressed in this species: CLOBB clustering formed a single group of actin gene as the biggest contig. Sequence alignment of the EST members showed that the actin gene of BPH had a coding capacity of 376 amino acid residues with a few single nucleotide polymorphisms among EST members. The actin gene is highly expressed in the thorax (TH and HT). This result is in accordance with the planthopper internal structure: the thorax is rich in muscle. The ESTs of the myosin gene, the eighth highly expressed gene, is also found mainly in TH and HT libraries.

**Figure 4:**
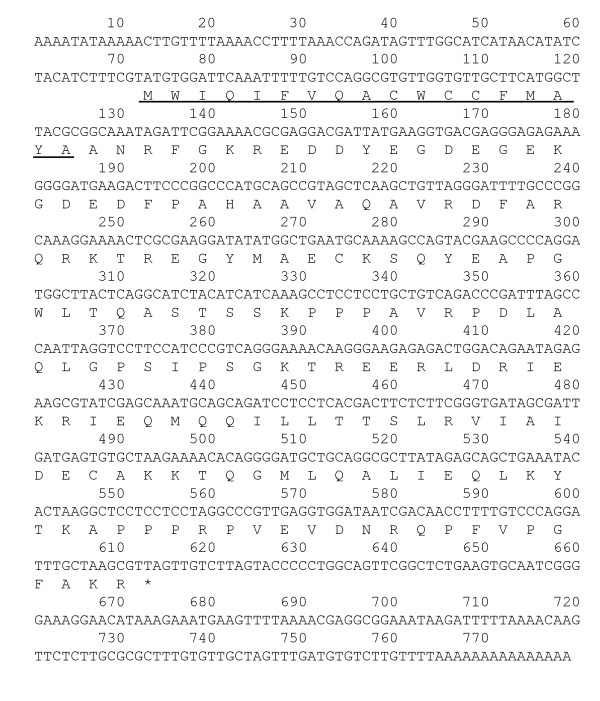
cDNA nucleotide sequence and putative deduced amino acid sequence of EST clone MB3851. The nucleotide sequence was determined based on the sequence alignment data of cluster members of MB3851. Stop codons are found at the upstream region of the putative first methionine. An InterProScan sequence search [27] showed a signal peptide in the first 18 amino acids (underlined). No homology was found in the DNA and protein databases.

**Figure 5:**
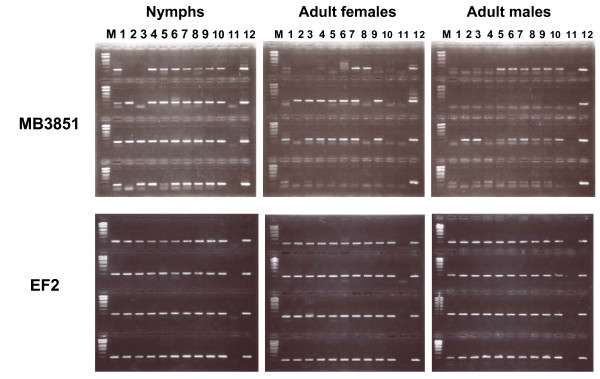
RT-PCR of EST clone MB3851 at various stages of BPH. PCR products amplified from 10 individual samples at each stage were electrophoresed on agarose gel. These samples were tested for positive amplification using a control PCR primer pair of elongation factor 2 gene (EF2) of BPH. Stage-dependent amplification was not clearly observed. Left panel: first row, 0-day-old third instar nymphs; second row, 1-day-old fourth instar nymphs; third row 0-day-old fifth instar nymphs; fourth row, 1-day-old fifth instar nymphs. Center panel: first row, 0-day-old female adults; second row, 1-day old female adults; third row, 2-day-old female adults; fourth row, 3-day-old female adults. Right panel: first row, 0-day-old male adults; second row, 1-day-old male adults; third row, 2-day-old male adults; fourth row, 3-day-old male adults. M, marker (lambda *Eco*T14I digest); 1–10, individual samples; 11, negative control of no template; 12, positive control.

A second abundantly expressed gene (MB3851 representative), which was mainly found in midgut libraries, showed no homology in the BLAST searches. This contig showed a coding capacity of a protein of 180 residues (Fig. 4). InterProScan [27] indicated that the first 18 amino acids corresponded to a signal peptide. More than 96% of ESTs belong to the male and female midgut libraries (MA and MB). Another five libraries, egg (EA and EB), 1st instar nymph (NA), salivary gland (SG), and testis (TA) libraries respectively contained this gene sequence in small numbers: six, four, one, five, and five. Subsequently, RT-PCR was performed to examine the expression stages of this gene in BPH. In all, 10 individuals of each stage (third instar nymph – 3-day-old adult) were tested. Gene expression, however, showed no uniformity among individuals and through the stages. Some individuals showed the PCR product and some did not (Fig. 5). Quantitative RT-PCR showed a parallel level of expression with standard ribosomal protein L4 in the ovary, midgut and testis (data not shown). Therefore, the RNA level of this gene seems to be high in BPH. The gene expression pattern was unusual. For that reason, genomic PCR was performed to examine whether this gene is coded in the BPH genome. However, we failed to obtain a PCR product from the genomic DNA using various combinations of 11 primers throughout the gene sequence and various amplification conditions. Genome-coded genes, such as the elongation factor 2 gene and ribosomal protein L4 gene, which are one-copy genes in *Drosophila melanogaster*, were amplified in similar conditions. This result suggests that the MB3851 gene is not in the BPH genome but rather came from an exogenous source. In fact, BPHs possess intracellular yeastlike symbiotes (YLS) in the fat body [28], but this gene was not found in the genome of YLS by a PCR-based survey. Moreover, the expression of this gene was observed in the tissues where YLS are not distributed. Bacteria in the gut do not also appear to be the source of this highly expressed gene because expression was not restricted to the gut. Moreover, genomic PCR from the intestine sample did not give a PCR product. A possible explanation is that this transcript or RNA derived from an exogenous source of BPH such as a virus or infectious RNA. Three virus species are known in BPH [20,22,23]; a different virus might be the source of this gene. Transmitted dsRNA replicons are often found in plants and fungi [29,30] and have been reported in insects [31].

Thirdly, an uncustomary gene, which was highly recovered from the salivary gland library (SG), was ranked. The gene product was rich in serine and alanine; some repeated amino acid sequences were found, suggesting that this gene product is a structural protein in BPH. This gene showed high homology to the gene expressed in the small brown planthopper, *Laodelphax striatellus*, which is annotated as mucin-like protein [accession number AAS88902]. Mucin is a heavily glycosylated protein forming a polymeric structure. It functions as mucosal barriers in some tissues. Planthoppers are vascular feeders and secrete coagulable saliva from the salivary glands for stylet feeding [32]. The protein of this gene product might be related to planthopper feeding. The predicted amino acid product, however, shows no homology with *Drosophila *mucin [AAF49596] [33].

Two enzyme genes, trypsin-like protease gene and enolase gene, were highly expressed in the planthopper. The protease was expressed in the midgut, showing expression in the libraries that contained the gut, AA, AB, MA, MB, NA, NB, and TH. About 97% of EST was recovered from the midgut libraries MA and MB. The trypsin-like protease apparently works for food digestion. This gene corresponds to AJ316142 reported in BPH [34]. On the other hand, enolase expression was found from a wide range of libraries, including the libraries of the salivary glands, ovary, and testis. Enolase is an essential glycolytic enzyme that catalyzes the conversion of 2-phosphoglycerate. Other highly expressed enzyme genes in BPH were ATP synthase subunit C (top 14th), arginine kinase (top 16th), and carboxylesterase (top 18th). The former two genes work for energy production. The carboxylesterase is related to insecticide resistance by catalyzing the insecticidal compounds [3,35].

Another notable gene was vitellogenin or vitellogenin-like protein gene. Results of CLOBB clustering showed that 238 ESTs belong to the vitellogenin gene cluster. The gene sequences of vitellogenin or vitellogenin-like proteins were not identical and were composed of several varied sequences. The fat body is the principal site of production of this yolk protein precursor in arthropods [36]. The expression of the vitellogenin gene, however, occurred throughout the female planthopper body; many ESTs (61 clones) were found in the salivary gland library (SG). No EST of vitellogenin was found in the libraries of the egg, nymphal, and male libraries in BPH. However, similar genes were found in male libraries: further precise analysis is necessary. The whitefly, *Bemisia tabaci*, a homopteran insect like BPH, shows much expression of vitellogenin in adults [15].

Two genes of low or no homology were ranked at seventh and ninth (Table 2). The gene AA0383 was found in libraries of the female gonad (OA, OB, OC) and the female abdomen (AA, AB), which contained the gonad. This result suggests that this gene is specifically expressed in the female gonad. This gene is the same as the female gonad-sepcifically expressed gene OC2756 described below. The cDNA size was short, about 500 bp, exhibiting the longest coding ability of 113 residues. The putative amino acid sequence was MSSKLFFVLATLALSALLSATESDAVYSNYALGSYGGYAYPSYAYPYYGYGYPYYSYGYPYYGYRYPYYSYGYPYYSYGYPYYSYGYPYYGYGGYGGYPYGGYGAYPGVGCVA, suggesting that this gene codes for a structural protein that is rich in tyrosine (39/113) and glycine (20/113). *In-situ *hybridization of this gene sequence showed specific expression in the lateral oviduct of the ovary as described later. Actually, BPH has swelled lateral oviducts, which become large and produce white material during maturation. This gene product seems to be a material that is secreted with oviposition.

The gene of no homology at ninth high expression in BPH (EST clone AB0581, 171 ESTs) was expressed in the midgut. In all, 167 ESTs were in the libraries of MA or MB, and each EST was found in AB, AC, NA, and NB libraries, whose samples contain the midgut. The specific expression in the midgut was confirmed using RT-PCR; no RT-PCR products were observed from the ovary and testis. This gene showed about 600 bp of cDNA size and coding capacity of 101 residues. The members of the EST cluster were composed of two variant sequences, especially in the 3'UTR. The two groups showed two amino acid differences in putative amino acid sequences, suggesting that they are variants of the same functional protein. Homology searches showed no related genes in the databases.

Ribosomal protein genes were also highly expressed. Ribosomal protein gene *RpS2 *was ranked at 5th and other ribosomal protein genes *RpL5 *and *RbL36 *were in the top 20 highly expressed genes.

### Expression of housekeeping genes among tissues – EST map

The gene expression pattern among libraries was visualized using a key word search in the BLASTX annotation and an Excel (Microsoft Corp.) macro program. This EST map appears to disclose the pattern of differential expression of highly expressed genes among tissues and stages. In the analyses of abundantly expressed genes in the previous section, abundance was judged from the direct number of ESTs in the libraries. In the EST map, a corrected value against the size of each library, the number of ESTs, was shown against 1,000 expressed genes. Although the ESTs that showed no key word in the highest score annotation text of BLASTX were not counted, the EST map provides a rough comparison of the expression pattern of a certain gene among tissues.

**Figure 6:**
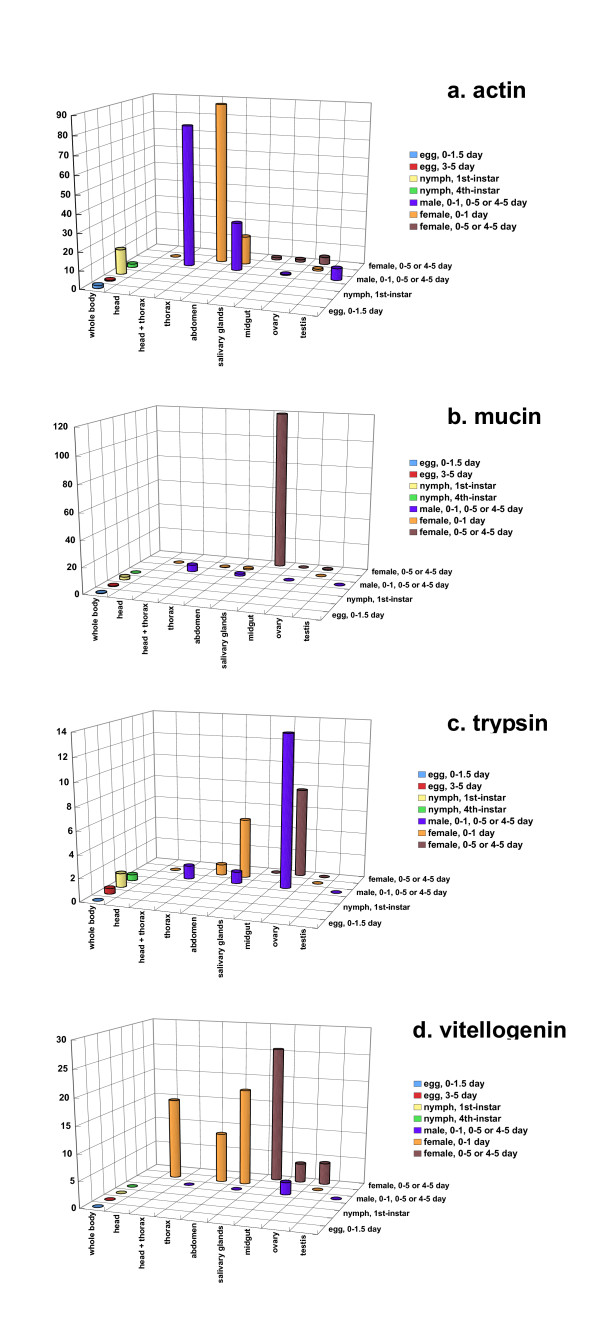
EST maps of highly expressed genes. The numbers of actin-, mucin-, trypsin-, and vitellogenin-related clones per 1,000 ESTs are shown as three-dimensional bar graphs. The libraries of the salivary glands, head, thorax, and ovary were made from female adults; those of head+thorax and testis were made from male adults. The libraries of the abdomen and midgut were made from both male and female adults, and the library of the whole body from nymphs. The stages shown in the right axis are 0–1.5 day of eggs, 3–5 day of eggs, 1st instar nymphs, 4th instar nymphs, adult males, 0–1 day adult females and 0–5 day adult females toward the far side.

Figure 6a shows the EST map of actin-related genes. The word "actin" was searched in the gene annotation text of the top-ranked annotation in each gene and the appearance frequency was counted in each library. The actin gene showed high expression in the thorax and abdomen. The abdomen was also the high expression site of the actin gene. A quite similar pattern of gene expression to that of the actin gene was observed in the myosin gene, which was the 8th highly expressed gene in Table 2. Mucin-like gene is mostly expressed in the salivary glands (Fig 6b). The expression was also observed in the head and thorax sample, which contained the salivary glands. Figure 6c was drawn using the key word "trypsin". Trypsin or trypsin-like protease gene was expressed in the tissues in which the midgut was included. However, the number of ESTs (y axis) was small in this gene; the key word "trypsin" might not be appropriate for EST map of this gene. The EST map of vitellogenin or vitellogenin-like genes showed high expression of the gene in females (Fig. 6d).

### Highly expressed genes in each body part and developmental stage

Abundantly expressed genes in each tissue or body part were examined. The top three contigs in the number of ESTs were selected using CLOBB analyses (Table 3). Most genes extracted in each library were so-called housekeeping genes. Some highly expressed genes showed no homology with the present data in the databases. An example of a body-part-specific gene was rhodopsin gene, which works for visualization and was the third in the number of ESTs in the female head (HA). The female thorax (TH) highly expressed actin and myosin; these genes appear to be expressed for the flight muscle, as described previously. Vitellogenin-like gene was the most abundant in the female abdomen (AA+AB). Vitellogenin is an egg yolk protein that is synthesized in the fat body before transportation into the eggs. Therefore, the high expression of this gene in female abdomen is reasonable. However, it was also found to be highly expressed in the salivary glands. Tissue specificity in gene expression was apparent and some specific genes were more abundantly recovered as ESTs than housekeeping genes. Functional analyses of these genes are important for elucidating specific features of BPH and probably homopteran or hemimetabolous insects.

**Table 3:** Top three genes highly expressed in each library

Library	Representative clone	No. of EST	Annotation
AA+AB	AA0013	32	gi|154799937|dbj|BAF75351.1| vitellogenin [Nilaparvata lugens] 352 1e-95
	AA0016	16	gi|90819992|gb|ABD98753.1| putative ADP/ATP translocase [Grap.. 261 4e-72
	AA1208	15	gi|51988476|gb|AAU20854.1| actin [Reticulitermes flavipes] 338 9e-92
AC+AD	AC1062	17	gi|110456520|gb|ABG74719.1| putative actin [Diaphorina citri] 421 e-116
	AC0157	11	n.h.
	AC0155	10	gi|50428904|gb|AAT77152.1| arginine kinase [Periplaneta america.. 285 6e-76
	AC0302	10	n.h.
	AC0779	10	gi|60678801|gb|AAX33735.1| MPA13 allergen [Periplaneta americ.. 198 3e-49
EA	EA0377	38	n.h.
	EA0026	36	n.h.
	EA0111	33	n.h.
	EA0258	16	gi|4504279|ref|NP_002098.1| H3 histone, family 3A [Homo sapiens] 263 1e-68
	EA0320	16	gi|124805775|ref|XP_001350534.1| eukaryotic translation initiatiati.. 115 1e-24
	EA0331	16	n.h.
EB	EB0055	91	n.h.
	EB0302	71	n.h.
	EB0169	54	gi|91078856|ref|XP_972007.1| PREDICTED: similar to 60S riboso.. 161 1e-38
HA	HA0029	54	gi|70909775|emb|CAJ17313.1| ribosomal protein L23Ae [Georissu.. 234 2e-60
	HA0105	44	n.h.
	HA0046	32	n.h. (homologous to rhodopsin)
HT	HT0003	223	gi|156640546|gb|ABU92560.1| actin [Monochamus alternatus] 171 2e-41
	HT0037	55	gi|56684881|gb|AAW22542.1| myosin light chain [Gryllotalpa orien.. 273 5e-72
	HT0065	52	gi|146229728|gb|ABQ12293.1| unknown [Antheraea pernyi nucleop.. 75 2e-12
MA	MA0011	371	n.h.
	MA0160	158	n.h. (homologous to trypsin-like protease)
	MA0065	108	n.h.
MB	MB0025	184	n.h.
	MB0037	91	n.h. (homologous to trypsin-like protease)
	MB0028	59	n.h.
NA	NA0013	130	gi|158286709|ref|XP_001688118.1| cuticular protein 140, RR-2 fa.. 101 3e-20
	NA0139	52	gi|46561736|gb|AAT01073.1| putative muscle actin [Homalodisca c.. 545 e-153
	NA1558	38	gi|158286709|ref|XP_001688118.1| cuticular protein 140, RR-2 fam.. 75 4e-12
NB	NB0017	87	n.h.
	NB0039	74	gi|91091510|ref|XP_969238.1| PREDICTED: similar to 60S riboso.. 373 e-102
	NB0029	62	gi|70909551|emb|CAJ17197.1| ribosomal protein S3e [*Scarabaeus.. *407 e-112
OA	OA0030	13	gi|110671482|gb|ABG81992.1| putative ribosomal protein S2 [Diap.. 164 2e-39
	OA0079	12	gi|157767586|ref|XP_001667273.1| Hypothetical protein CBG1604.. 79 7e-14 (ovary-specifically expressed OC2756 in Table 4)
	OA0009	11	gi|155966151|gb|ABU41030.1| hypothetical protein [Lepeophtheirus.. 89 2e-16
OB+OC	OB0024	210	gi|157767586|ref|XP_001667273.1| Hypothetical protein CBG16043.. 82 2e-14 (ovary-specifically expressed OC2756 in Table 4)
	OB0360	118	gi|93102305|dbj|BAE93436.1| shematrin-4 [Pinctada fucata] 97 7e-19 (ovary-specifically expressed OC0606 in Table 4)
	OB0055	40	gi|82470775|gb|AAL87229.3|AF480890_1 metacaspase [Acantham.. 88 4e-16
SG	SG0085	490	gi|46360158|gb|AAS88902.1| mucin-like protein [Laodelphax striat... 222 2e-56
	SG0019	108	gi|154799937|dbj|BAF75351.1| vitellogenin [Nilaparvata lugens] 404 e-111
	SG0023	107	gi|6523547|emb|CAB62280.1| hydroxyproline-rich glycoprotein DZ-.. 110 4e-23
	SG0076	107	n.h.
TA	TA0775	19	n.h. (testis-specifically expressed NB3697 in Table 5)
	TA0186	15	n.h.
	TA0012	12	n.h. (testis-specifically expressed AD1216 in Table 5)
	TA0118	12	gi|66525050|ref|XP_625145.1| PREDICTED: similar to tubulin, alph.. 285 1e-75
	TA0428	12	gi|110755210|ref|XP_393150.3| PREDICTED: similar to dumpy CG.. 71 6e-11
TH	TH0004	402	gi|51988476|gb|AAU20854.1| actin [Reticulitermes flavipes] 469 e-130
	TH0054	117	gi|17933672|ref|NP_524586.1| Myosin LC2 [Drosophila melanogas.. 221 5e-56
	TH0020	62	gi|154799937|dbj|BAF75351.1| vitellogenin [Nilaparvata lugens] 395 e-108

Representative clones are shown by an EST number in the cluster. "n.h." denotes no homology. HA0046 showed no homology, but other members of the cluster showed homology to rhodopsin. Other cluster members of MA0160 and MB0037 showed homology to trypsin-like protease.

### Genes specifically expressed in the gonads

Reproductive organ-specifically expressed genes were sought. Insects showed various types of reproduction. Elucidation of the function of genes specifically expressed in the gonads is an interesting subject of research and an important topic for insect management through reproductive suppression or stimulation. Planthoppers, which are hemimetabolous insects, have telotrophic ovarioles, whereas holometabolous insects, such as fruit flies, mosquitoes, honeybees, and silkworms, have polytrophic ovarioles. Specific genes or specific function in the hemimetabolous insects are expected in the gonad-specifically expressed genes of BPH.

**Figure 7:**
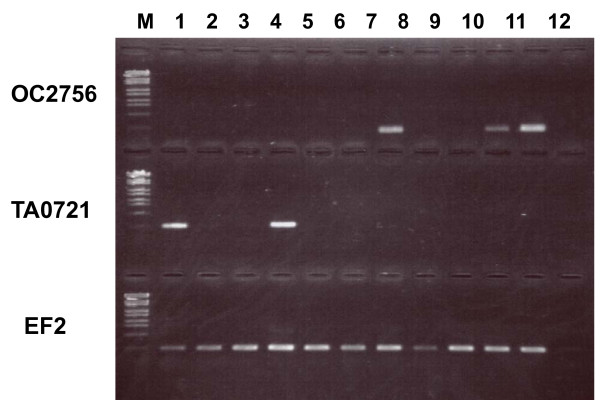
RT-PCR of EST clones OC2756 (AA0383) and TA0721 showing specific expression in the gonads. EF2, elongation factor 2 gene (control); M, marker lambda-*Eco *T14I digest; 1, male abdomen; 2, male head; 3, male midgut; 4, testis, 5, 2nd instar nymph; 6, 4th instar nymph, 7, female abdomen; 8, female head; 9, female midgut; 10, ovary of 0-day-old adult; 11, ovary of 4-day-old adult; 12, negative control of no template.

**Figure 8:**
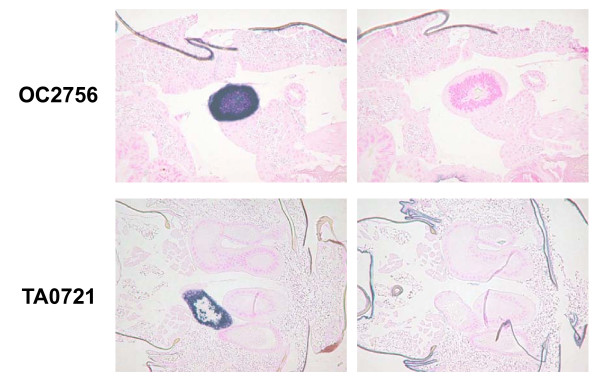
*In situ *hybridization of EST clone OC2756 (AA0383) in adult female and TA0721 in adult male. Left, anti-sense probe; right, sense probe. Signals were observed in a part of the lateral oviduct (OC2756) and in an accessory gland of the testis (TA0721).

In this species, the ovary starts to mature one or two days after adult emergence. Library OA is made from the immature ovary and its accessory glands of 0–1 day old adults; OB and OC were made from mature ones of 4–5 days old. Libraries AA and AB were made from the female abdomen, which includes the female gonad. Therefore, the ESTs found only in the libraries OA, OB, OC, AA, and AB are candidate genes for female-gonad-specific expression. Table 4 shows the example of candidate ESTs of female-gonad-specifically expressed genes. These genes were selected by high-frequency expression in these five libraries and no expression in the other libraries. The ESTs that show expression in the libraries OB and OC and no expression in the library OA (e.g. EST clones AB0551, OB0998, OB1061, OC0638, OC0681) were probably expressed in the mature ovary and accessory glands. The restricted expression of these genes in the female gonad was confirmed by RT-PCR. Figure 7 shows the restricted gene expression of EST clone OC2756 in the female gonad and abdomen. *In situ *hybridization showed expression of this gene in the lateral oviduct (Fig. 8) as described previously. Further examination is necessary for characterizing these genes in ovarian development of BPH.

**Table 4:** Genes specifically expressed in the female gonad

Representative clone	No. of ESTs in each library							Homology search BLASTX
		
	Total	OA	OB	OC	AA	AB	Others	
AB0551	24	0	12	10	1	1	0	gi|7657285|ref|NP_056624.1| keratin associated
	24	0	12	10	1	1	0	protein 5-4 [Mus... 128 2e-28
OB0570	10	0	3	5	1	1	0	gi|7106224|gb|AAF36090.1| flagelliform silk
	38	8	5	19	2	4	0	protein [Nephila clavipes] 75 4e-12
OB0998	15	0	7	7	0	1	0	n.h.
	14	0	7	7	0	0	0	
OB1013	27	2	9	16	0	0	0	gi|24585618|ref|NP_724320.1| CG31626
	26	1	9	16	0	0	0	CG31626-PA, isoform A [Dros... 136 1e-30
OB1061	10	0	9	1	0	0	0	n.h.
	10	0	9	1	0	0	0	
OC0606	122	8	30	76	5	3	0	gi|93102305|dbj|BAE93436.1| shematrin-4
	121	9	30	78	1	3	0	[Pinctada fucata] 97 7e-19
OC0638	20	0	9	11	0	0	0	gi|149258154|ref|XP_001480786.1| PREDICTED
	20	0	9	11	0	0	0	hypothetical protein... 159 2e-37
OC0681	35	0	17	18	0	0	0	gi|82880761|ref|XP_901600.1| PREDICTED:
	35	0	17	18	0	0	0	hypothetical protein LOC... 161 2e-38
OC1014	42	6	4	26	4	2	0	gi|155966143|gb|ABU41026.1| hypothetical
	38	6	4	26	0	2	0	protein [Lepeophtheirus... 83 2e-14
OC1083	27	2	7	18	0	0	0	gi|72547036|ref|XP_843163.1| proteophospho-
	26	1	7	18	0	0	0	glycan 5 [Leishmania ma.. 72 4e-13
OC2756	119	6	26	85	0	2	0	gi|157767586|ref|XP_001667273.1| Hypothetical
	228	12	55	155	3	3	0	protein CBG16043 [Caeno... 79 9e-14

The numbers of ESTs detected in the five libraries related with the ovary and its accessory glands are shown. OA, OB and OC are the libraries from the female gonad and AA and AB are those from the female abdomen. The upper line is the number of cluster members in each library using the clustering algorithm CBP; the lower line those using CLOBB. "n.h." denotes no homology. The category for 'no homology' is described in Gene annotation in *Methods*.

Male-gonad-specifically expressed genes are presented in Table 5. Genes expressed in the testis and its accessory glands should be in the library TA and the libraries of the male abdomen AC and AD, in which the male gonad is included. Since the male gonad starts to develop from the senior nymphal stage, the library NB might also include the gonad-specifically expressed genes. Therefore, the genes expressed in these libraries and not in other libraries were nominated. Candidate genes were brought into RT-PCR examination and the genes confirmed to be expressed only in the male gonad are shown in Table 5. The results of RT-PCR and *in situ *hybridization of EST clone TA0721 are portrayed respectively in Fig. 7 and Fig. 8. TA0721 was transcribed in an accessory gland of the testis.

**Table 5:** Genes specifically expressed in the male gonad

Representative clone	No. of ESTs in each library						Homology search BLASTX
		
	Total	TA	AC	AD	NB	Others	
AC0657	11	7	4	0	0	0	n.h.
	11	7	4	0	0	0	
AC0743	6	4	1	1	0	0	gi|157133853|ref|XP_001663041.1| hypothetical
	9	5	1	3	0	0	protein AaeL_AAEL02908.. 72 2e-11
AC0825	10	6	3	1	0	0	gi|114629259|ref|XP_521410.2| PREDICTED:
	10	6	3	1	0	0	hypothetical protein [Pan.. 100 2e-19
AD1216	17	12	2	3	0	0	n.h.
	17	12	2	3	0	0	
NB2328	17	11	2	2	2	0	n.h.
	17	11	2	2	2	0	
NB3697	26	19	1	3	3	0	n.h.
	26	19	1	3	3	0	
NB5623	9	4	2	0	3	0	n.h.
	19	11	2	2	4	0	
TA0293	11	10	0	1	0	0	gi|15613784|ref|NP_242087.1| hypothetical
	11	10	0	1	0	0	protein BH1221 [Bacillus.. 72 3e-11
TA0428	6	6	0	0	0	0	gi|110755210|ref|XP_393150.3| PREDICTED:
	17	12	3	2	0	0	similar to dumpy CG3319.. 71 6e-11
TA0528	12	10	0	2	0	0	n.h.
	11	9	0	2	0	0	
TA0721	20	11	1	8	0	0	n.h.
	20	11	1	8	0	0	
TA1050	4	2	0	2	0	0	gi|91084421|ref|XP_968215.1| PREDICTED:
	14	9	0	5	0	0	similar to CG6414-PA [Tribo.. 116 1e-24
TA1095	12	8	2	2	0	0	n.h.
	12	8	2	2	0	0	

The numbers of ESTs detected in the four libraries related with the testis and its accessory glands are shown. TA is the library of the male gonad, AC and AD are those from the male abdomen, and NB is that of 4th instar nymphs. The upper line is the cluster member in each library using the clustering algorithm CBP; the lower line those using CLOBB. "n.h." denotes no homology. The category for 'no homology' is described in Gene annotation in *Methods*.

The genes listed in Tables 4 and 5 are some gonad-specifically expressed genes. These genes were able to be selected because they expressed highly in the libraries of the gonad or the body part containing the gonad. Although this approach to extract tissue-specifically expressed genes is useful, it is not easy to obtain genes that are not actively expressed in the tissues.

### General feature of EST libraries and Gene ontology analysis

**Figure 9:**
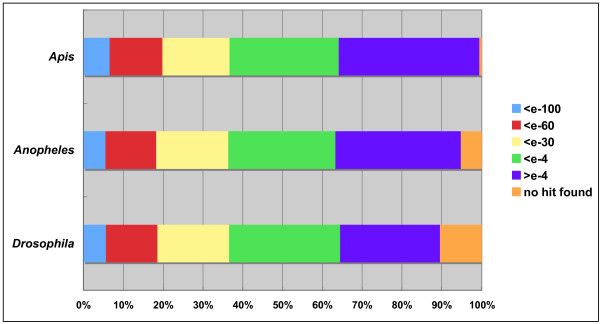
Homology of planthopper ESTs with three insect species by BLASTX. All 37,122 ESTs were examined against protein databases of *Apis mellifera*, *Anopheles gambiae*, and *Drosophila melanogaster*. The ratios of the EST clones are shown based on the *e*-value in the annotation description of BLASTX.

Insects are a large group among animals and are quite divergent. Many ESTs of BPH showed no homology in the nucleotide and amino acid sequences. To examine the similarity level of the genes among insect species, we conducted homology searches using BLASTX against protein data of three insect species, *Drosophila melanogaster*, *Anopheles gambiae*, and *Apis mellifera*. All 37,122 ESTs were examined against each protein database, and were grouped by the e-value shown in the BLAST result (Fig. 9). Of ESTs, 35–40% showed homology with the proteins of the three insect species with <*e*-30, but 35–40% showed very little or no homology with the proteins of the three insects. Some genes showing 'no homology' or 'no-hit-found' might be those that are not translated into proteins. It is worth consideration that BPH is a hemimetabolous insect, which has no pupal stage, and the other three species are holometabolous ones. This difference might affect the low homology between them.

## Conclusion

The most feared pest of rice plants is BPH. The EST database constructed in this study and described herein will be useful for the molecular studies of BPH. BPH shows resistance against various insecticides. Insecticide resistance mechanisms are considered to be attributable to the high enzyme activities, which metabolize the compounds quickly [3,35,37]. They are also partially attributable to the mutations in the genome sequences in the target molecules, which cause insensitivity to the chemicals [4,38]. Gene sequences of the metabolic enzymes and insecticide-target molecules might be obtained from the database. The EST database is also useful for identifying potential target genes for developing novel insecticides against planthoppers [39]. The database will also be useful for finding important functional molecules [34,40,41] as well as for searching for hemipterous insect-specific or tissue-specific genes. Genomic sequence data are unavailable in *Nilaparvata lugens*. Therefore, EST data will also be used for identification of proteins in proteomic studies [42].

Further determination of complete cDNA sequences is underway. Microarrays produced based on the EST data and some additional sequences from ongoing full-length cDNA analyses are expected to become powerful tools for gene expression analyses. The microarray analyses will be applied for elucidation of mechanism of wing dimorphism in planthoppers. Long or short wings are induced by environmental stimuli during nymphs and the response is different between sexes. A recently developed molecular sexing method in BPH [43], which enable to distinguish sexes in nymphal stages, will contribute to studying wing dimorphism in BPH together with the microarrays. Gene expression profiles of BPH that attack various resistant varieties of rice plants will be useful for elucidating the molecular mechanism of rice plant resistance and planthopper virulence against resistant rice plants. Expressed genes related to plant-sucking and nutrient digestion are also important targets in BPH physiological and biochemical studies. In addition, results reveal that RNA interference (RNAi) is quite effective in BPH (data in preparation). This important agricultural pest might also be used as a hemimetabolous model insect in the near future as well as several aphid species. The continued accumulation of information related to *Nilaparvata lugens *can promote efforts to control other economically important rice planthopper species: *Sogatella furcifera *and *Laodelphax striatellus*.

## Methods

### Insects and RNA preparation

*Nilaparvata lugens *(Hemiptera, Delphacidae) were collected in Izumo, Shimane Prefecture, Japan in 1987. This Izumo strain was reared with rice seedlings at 26°C under a long day photoperiod of 16 h of light and 8 h of dark in plastic boxes as a stock population.

Egg samples were collected from 5% sucrose solution into which mature females were allowed to lay eggs through a proprietary membrane (Parafilm M; American National Can Group Inc., Chicago). Nymphs were reared in glass bottles (ca. 18 cm high; 9 cm diameter) containing rice seedlings. Stage-specific samples were prepared from those reared in a test tube (ca. 13 cm high; 16 mm diameter) with rice seedlings. Approximately 500 insects were used for lambda ZAP libraries and ca. 50 for pGEM plasmid libraries. More than 1,000 individuals were used for egg and young nymphal libraries. Planthopper tissues were dissected under a binocular. The planthoppers were dipped in 70% ethanol for several seconds and washed in 0.85% NaCl solution. They were then dissected in a droplet of saline solution with fine forceps and the tissues were dipped into the extraction buffer of QuickPrep Micro mRNA Purification Kit (Amersham Biosciences UK Ltd., England) and polyA+ mRNA was purified. Some large samples were homogenized in the extraction buffer in a microfuge tube using a small pestle.

### cDNA Libraries

Two libraries were made for tissues. One was that made with lambda-ZAPII (Stratagene, USA). The cDNAs were inserted unidirectionally into the lambda ZAP II vector and *in vivo *mass excision of the pBluescript SK phagemid from the ZAP vector was performed to obtain bacterial colonies. The other is a plasmid library of amplified cDNA. First strand cDNA was synthesized using an oligo-dT primer (5'-TGTGTCTAGAGGATCCGTACCCAGC(T)_30_VN-3') along with SMART II oligonucleotide (5'-AAGCAGTGGTAACAACGCAGAGTACGCGGG-3') in a SMART RACE cDNA amplification Kit (Clontech Laboratories Inc., USA). Amplification of cDNA was performed with SMART technology using the nested universal primer (NUP) (5'-AAGCAGTGGTAACAACGCAGAGT-3') in the kit and 3'-PCR primer (5'-TGTGTCTAGAGGATCCGTACCCAGC-3'). The amplified DNA was purified with sepharose CL-2B (Amersham Biosciences AB, Sweden) in a mini-column (60 × 7.5 mm; about 2.5 ml) to eliminate small sized DNA and ligated into pGEM-T (Promega Corp., USA) by TA-cloning. Usual blue-white selection of the bacterial colonies was done on the LB agar plates.

### Sequencing

Sequencing templates were prepared by colony PCR from bacterial colonies on the agar plate using M13–20 and reverse primers. The amplified products were purified with a spin column of sephacryl S-300 HR (Amersham Biosciences AB, Sweden). The sequencing reaction was performed using Big Dye Terminator v3.0 or v3.1 (Applied Biosystems, USA) with a DNA Sequence System (model 377, PE Applied Biosystems) or a DNA analyzer (model 3700, PE Applied Biosystems). The sequencing primers were SK primer 5'-CGCTCTAGAACTAGTGGATC-3' for the clones in pBluescript, and NUP primer for the clones in pGEM. These primers enable us to read the sequence of the positive strand.

### Sequence Processing

All sequences generated from the sequencers were first processed manually to remove vector sequences and improper regions of the sequence. In order to delete contaminants and unnecessary sequences in the ESTs, a local BLAST search using the BLASTALL program downloaded from NCBI [44] was performed with a desktop computer (Mac OSX; Apple Computer Inc., USA). Planthopper EST database file was made using Formatdb program and BLASTN searches were performed. The following genome or gene sequences were eliminated: cloning vectors (lambda ZAP II and pGEM), *Escherichia coli *genome, planthopper viruses [NLRV, NC_003652–NC_003661; NLCXV, AB183424, himetobi P virus or Hi-PV, AB183472]; planthopper mitochondrial genome; and planthopper partial ribosomal rRNA gene including retrotransposon R2. The EST sequences showing a homology E-value of <1.0 × 10^-20 ^to the query sequences were eliminated from the EST libraries. The ESTs were further tested for vector sequence contamination using NCBI VecScreen; ESTs of less than 300 bp were also discarded.

### EST Assembly

The ESTs were grouped into clusters using two clustering algorithms. One is the Cluster on the basis of BLAST similarity (CLOBB) [25]. The parameters used in this Perl script were 95% identity and 50 bp coverage. The other is the method of combined BLAST and PhredPhrap (CBP) [26]. In this method, the identity value and coverage length were started from 97% and 90 bp, respectively; the coverage length value and identity value were raised to attain the final consensus result. The contig members were tested for a 95% identity and 90% covering ratio after the clustering process.

### Gene annotation

Genes were identified by sequence similarity comparison against NCBI RefSec using BLASTX. We used the -F F option for examining low complexity sequences. The degree of sequence homology was based on the following standards. 'High homology' is given to the EST clones which fulfilled the following three conditions: E-value is 1e-10 or less, amino acid homologous regions were more than 99 bp, and the identity is 30% or more. 'Low homology' signifies that the E-value is 1e-10 or less, but the two other conditions described for the high homology categories are not fulfilled. 'No homology' fulfilled no conditions.

### EST map

BLAST annotation text was used to visualize the expression pattern of housekeeping genes among libraries. The BLASTX searches were performed in 37,122 ESTs and the top annotation description for each EST was selected in the text-base in individual libraries. A key word search was done through the text of all selected annotation descriptions in each library, and the hits was counted. The number of hits was considered as the number of cDNA related to the key word. The numbers of ESTs analyzed differ among libraries. For that reason, the number of hits was normalized by conversion into the number per 1,000 ESTs (number of hits/number of EST in each library × 1,000). The normalized value (number of hits per 1,000 ESTs) was visualized using three-dimensional bar charts produced using Excel software (Microsoft Corp., USA).

### BLASTX analysis against insect amino acid databases

BLASTX analyses were performed for all ESTs as query data against amino acid databases of *Drosophila melanogaster*, *Anopheles gambiae*, and *Apis mellifera*. Protein sequence databases of these insect species were obtained from NCBI. Database sizes of *D. melanogaster*, *A. gambiae*, and *A. mellifera*, were, respectively, 76,066, 33,024, and 9,965 proteins or amino acid sequences. The BLAST command used was "blastall -p blastx -F F -v 1 -b 1 -m 8". The BLAST results were divided into six groups according to the *e*-value, <*e*-100, <*e*-60, <*e*-30, <*e*-4, > *e*-4, and no hit found.

### RT-PCR

A highly expressed gene, MB3851, and gonad-specifically expressed genes were examined for transcriptional specificity in stages or tissues. Messenger RNA of desired stages or tissues were extracted using an RNeasy mini kit (Qiagen Inc., USA); cDNA was synthesized using the ExScript RT reagent Kit (Takara Bio Inc., Japan) with oligo-dT primer or random 9-mer primers. Thermal conditions for RT-PCR were usually 95°C for 10 sec, followed by 30–35 cycles of 95°C for 30 sec, 52°C for 30 sec, and 72°C for 1 min. Primers were designed based on contig sequences generated from ESTs of the same cluster (Table 6). The PCR products and their sizes were examined using agarose gel electrophoresis followed by ethidium bromide staining. The prepared cDNAs were tested for validity of template preparation using a primer pair of the elongation factor 2 gene of BPH.

**Table 6:** Primer sequences used for RT-PCR

Representative clone		Primer sequence	Product size (bp)
Highly expressed in BPH			
MB3851	forward	GGCGTGTTGGTGTTGCTTCA	515
	reverse	GCTTAGCAAATCCTGGGACA	
			
Female gonad-specifically expressed			
AB0551	forward	AATCATCGCTGCTTTCCTCGCC	437
	reverse	GGGAGCAAGGAGAGGCTATTAC	
OB0570	forward	GGGACACAGACTTAACTTGGCG	505
	reverse	AGCAACTCTTGCTCCATAGT	
OB0998	forward	TCCCAAAGTCGATGGCCGAATC	529
	reverse	TGGTACTGACTGATCGTTCC	
OB1013	forward	GACGGAGCAACCGTACATAT	486
	reverse	CGGTGAAGTAGATGGTCTCA	
OB1061	forward	CATCAGCCCTGTGACGATAGAG	602
	reverse	TCTGCACTCGCTGCTGTTCCAT	
OC0606	forward	CTGTGATCTCAAGCTCAGCCTG	498
	reverse	CACCAGCATGACCTTGGAGTAC	
OC0638	forward	GTCTGTGCCCTTTGCGCCAT	504
	reverse	GCATGGCTGATGGCAGACTG	
OC0681	forward	GTAAAATGGCCGCCAAGTCACT	435
	reverse	GAATGGAGAGGTGTGTTGCA	
OC1014	forward	CGTTGTTGCTGCCTTGGCACTA	480
	reverse	CCTGGATTTGCCTAAGCTGCAA	
OC1083	forward	CATCCTGTGGATGCGGATACTC	512
	reverse	GACTAGTAAAGCACAGATCG	
OC2756	forward	AGTGATCACAAGCTCTGCCTG	382
	reverse	TATGCCACACAGCCAACACCAG	
			
Male gonad-specifically expressed			
AC0657	forward	CGAGCTCTGTGTTGCTGAAG	575
	reverse	CAGCAGGAGTTAAAACATGGG	
AC0743	forward	CACCAACTCAACACTCCAC	525
	reverse	TTACTTCGGCCACACGGCTC	
AC0825	forward	GCGACTGGTGCAAAGTAGCA	610
	reverse	TCTTGACTCAGCTTCCCATC	
AD1216	forward	CTTCATTGTGGCTGTTGCAAG	444
	reverse	TCAGGGTATAGTGACCTTCC	
NB2328	forward	CCTCAGTACACAGTGAAACTC	480
	reverse	GCTCTTCACAGGGATCCACA	
NB3697	forward	GGCAGAATCAGTCGAAGATC	435
	reverse	GCGCCACATTTTGGAATGGG	
NB5623	forward	AGAACCAAACCCAAACAGGC	416
	reverse	GTTACACGGATACAAACCCTG	
TA0293	forward	ACCATGCAGCAGAGGGAATC	542
	reverse	GGATGGGTGACCATGGAGTC	
TA0428	forward	CCATCTCCTGAAACTGTAGG	464
	reverse	AGGCAGTTCAAGTGGCTCTC	
TA0528	forward	CCAAAACTCCTACGGTACCG	502
	reverse	TTTTTCGCAGGGGTCGGTGC	
TA0721	forward	CACTCATCTCCAGTGATACC	452
	reverse	CTGGTGCTAATGTTGGAGCG	
TA1050	forward	TCATTTTCCCGATGGCAAGC	527
	reverse	TTTCGACGGCACGCTTCTCC	
TA1095	forward	CCAGAGATCCTGCAGAGACA	545
	reverse	GCCTTAGTGTAACACTATTCCC	
			
Control PCR primer for template validation			
NL_EF2	forward	GAAGTTCAGTGTGTCGCCTG	420
	reverse	TCTCCCAGATATCTGGCTCT	

### *In situ *hybridization

Planthopper specimens for *in situ *hybridization were fixed with 5% formaldehyde including 0.5% picric acid and embedded in paraffin wax. The tissue sections were de-waxed with xylene and re-hydrated through an ethanol series. The sections were fixed with 4% paraformaldehyde in phosphate-buffered saline (PBS) and treated with 10 mg/ml Proteinase K for 30 min at 37°C. After re-fixing with the 4% paraformaldehyde, they were acetylated by incubation in 0.1 M triethanolamine-HCl, pH 8.0, 0.25% acetic anhydride for 10 min. After dehydration through an ethanol series, hybridization was performed with probes at concentrations of 100 ng/ml in the Probe Diluent (Genostaff, Tokyo) at 60°C for 16 hr. About 350 bp fragment DNA were made using PCR, and digoxigenin (DIG)-labeled sense and anti-sense probes were produced by DIG RNA Labeling Kit (Roche Diagnostics Corporation). After hybridization, they were washed in 5× SSC, at 60°C for 20 min and then in 50% formamide, 2× SSC at 60°C for 20 min, followed by RNase treatment for 30 min at 37°C. The sections were then washed twice with 2× SSC, 0.2× SSC, and 0.1% Tween in Tris buffered saline (TBST). After treatment with 0.5% blocking agent (Roche) in TBST, the sections were incubated with anti-DIG AP conjugate (Roche) diluted 1:1000 with TBS for 2 hr. Coloring reactions were performed with BM purple AP substrate (Roche). The sections were counterstained with Kernechtrot stain solution (Mutoh Chemical Co., Tokyo).

## Abbreviations

BLAST: Basic local alignment search tool; BPH: The brown planthopper; CBP: Method of combined BLAST and PhredPhrap; CLOBB: cluster on the basis of BLAST similarity; EST: expressed sequence tags; GO: gene ontology; Hi-PV: himetobi P virus; NCBI: National Center for Biotechnology Information; NLCXV: Nilaparvata lugens commensal X viurs; NLRV: Nilaparvata lugens reovirus; RT-PCR: reverse transcriptase-polymerase chain reaction; SMART: switching mechanism at 5' end of the RNA transcript.

## Authors' contributions

HN participated in the conception of the project, experimental design, sample preparation, data analyses, and manuscript preparation. SK studied gonad-specifically expressed EST and conducted RT-PCR. YK performed plasmid library construction, EST sequencing and manual processing of the sequence data. KM constructed phage libraries. QZ participated in sequencing and data processing of some clones. SF performed EST annotation and constructed a web site for the EST database. MS performed EST clustering by CBP and constructed EST map. KM provided overall knowledge related to the EST project. All authors read and approved the final manuscript.
